# High class I HDAC activity and expression are associated with RelA/p65 activation in pancreatic cancer *in vitro *and *in vivo*

**DOI:** 10.1186/1471-2407-9-395

**Published:** 2009-11-13

**Authors:** Annika Lehmann, Carsten Denkert, Jan Budczies, Ann-Christin Buckendahl, Silvia Darb-Esfahani, Aurelia Noske, Berit Maria Müller, Marcus Bahra, Peter Neuhaus, Manfred Dietel, Glen Kristiansen, Wilko Weichert

**Affiliations:** 1 Institute of Pathology, Charité University Hospital, Berlin, Germany; 2 Department of General, Visceral, and Transplantation Surgery, Charité University Hospital, Berlin, Germany; 3 Institute of Surgical Pathology - University Hospital, Zurich, Switzerland

## Abstract

**Background:**

The strong association between aberrant HDAC activity and the occurrence of cancer has led to the development of a variety of HDAC inhibitors (HDIs), which emerge as promising new targeted anticancer therapeutics.

**Methods:**

Due to the pivotal role of RelA/p65 in the tumorigenesis of pancreatic neoplasia we examined the expression of class I HDACs 1, 2 and 3 in a large cohort of human pancreatic carcinomas and correlated our findings with RelA/p65 expression status. Furthermore, we investigated the impact of the HDIs SAHA and VPA on RelA/p65 activity in pancreatic cancer cell culture models.

**Results:**

Class I HDACs were strongly expressed in a subset of pancreatic adenocarcinomas and high expression was significantly correlated with increased nuclear translocation of RelA/p65 (p = 0.024). The link of HDAC activity and RelA/p65 in this tumor entity was confirmed *in vitro*, where RelA/p65 nuclear translocation as well as RelA/p65 DNA binding activity could be markedly diminished by HDI treatment.

**Conclusion:**

The RelA/p65 inhibitory effects of SAHA and VPA *in vitro *and the close relationship of class I HDACs and RelA/p65 *in vivo *suggest that treatment with HDIs could serve as a promising approach to suppress NF-κB activity which in turn may lead to enhanced apoptosis and chemosensitization of pancreatic cancers.

## Background

Posttranslational modifications such as acetylation and deacetylation of histone proteins play an important role in chromatin remodelling and transcriptional regulation [1]. Two groups of corresponding enzymes, histone deacetylases (HDACs) and histone acetyl-transferases (HATs) act in concert to maintain the balance between condensed and relaxed chromatin by catalyzing the removing or adding of acetyl groups to specific lysine-rich amino terminal histone residues. Chromatin condensation due to high HDAC activity leads to transcriptional silencing of a subset of genes involved in differentiation and inhibition of proliferation, apoptosis and metastasis [2]. Furthermore, HDACs are able to directly deacetylate tumor relevant non-histone proteins such as p53, GATA-1, β-catenin and NF-κB (RelA/p65) [3], which may alter their activity, subcellular localization and interaction partners.

In humans, four structurally diverse classes of HDACs comprising 18 isoforms have been identified so far with class I HDACs 1, 2 and 3 being the best characterized and most abundantly expressed isoforms in tumor tissues [4].

Due to the fact that aberrant HDAC activity has been associated with the occurrence of different types of cancers [5,6], a variety of clinically applicable HDAC inhibitors (HDIs) have been developed and tested during the past few decades [3,7-10].

HDIs have shown to suppress tumor growth and to induce differentiation and apoptosis in various studies both, *in vitro *and *in vivo *[2,11]. Some of them including suberoylanilide hydroxamic acid (SAHA) and valproic acid (VPA) are in late-phase clinical trials for the treatment of solid tumors and show promising effects with low toxicity [3]. Recently SAHA was approved by the Food and Drug Administration for the clinical use in patients with cutaneous T-cell lymphoma [12].

Pancreatic adenocarcinoma is the fourth leading cause of cancer death in the United States. Due to the high chemoresistance and the fact that only 5-28% of pancreatic carcinomas are surgically resectable at the time of diagnosis the possibilities of curative therapy are highly restricted. Thus, 5-year survival rate is lower than 5% [13]. New strategies for the treatment of pancreatic carcinoma, particularly with regard to the avoiding of chemoresistance are urgently needed [14].

Reduced sensitivity to chemotherapeutic agents is often associated with a constitutive active Rel/NF-κB pathway [15,16]. The Rel/NF-κB family consists of various members of transcription factors, p50/p105 (NF-κB1), p52/p100 (NF-κB2), c-Rel, RelB and p65 (RelA), which are responsible for the regulation of immune and inflammation related genes such as cytokines, cytokine-receptors and cell adhesion molecules [17]. Overexpression and/or dysregulation of certain regulatory proteins of the NF-κB pathway, e.g. the heterodimer p65/p50, have been linked to higher tumor grade and poor prognosis in consequence of increased cell proliferation, angiogenesis and metastasis [18,19].

NF-κB activation can be regulated at several levels. In resting cells, inactivated NF-κB is sequestered in the cytoplasm by the inhibitory factor IκBα. In response to specific pro-inflammatory signals such as tumor necrosis factor-α (TNF-α) and interleukin-1β (IL-1β), IκBα becomes phosphorylated, ubiquitinylated and subsequently degradated allowing a rapid nuclear translocation and thereby activation of NF-κB [17]. Apart from translocation based activation, NF-κB can be regulated by proteolytic procession or posttranslational modifications like HDAC mediated acetylation or deacetylation, suggesting a potential RelA/p65 inhibitory effect of HDIs like SAHA and VPA [20].

In this study we, for the first time, investigated the expression of class I HDACs in a large cohort of human pancreatic carcinomas. Due to the pivotal role of RelA/p65 in the tumorigenesis of pancreatic carcinoma we correlated our findings with RelA/p65 expression status. Based on the fact that RelA/p65 is a putative target of HDIs, we additionally tested the effects of the two well known HDIs SAHA and VPA on RelA/p65 activity *in vitro*.

## Methods

### Study Population

Tissue samples from 81 patients who underwent partial pancreaticoduodenectomy for primary pancreatic ductal adenocarcinoma at the Charité University Hospital between 1991 and 2000 were used in this study. The study has been approved by the Charité University Ethics Committee under the title "Retrospektive Untersuchung von Gewebeproben mittels immunhistochemischer Färbung und molekularbiologischer Methoden" ("*Retrospective analysis of tissue samples by immunohistochemistry and molecular biological techniques*"; EA1/06/2004) at the 20^th ^of September 2004.

Median age of patients with pancreatic cancer was 66 years (range 39-80 years). Follow-up data regarding overall survival were available for all patients. Within the follow up time, 64 patients (79%) died after a median follow up time of 11.7 months. Median follow-up time of patients still alive at the endpoint of analysis was 44.0 months. Cases were staged according to "TNM Classification of Malignant Tumours. 6th edition" (Sobin LH, Wittekind C.; 2002) and cases were graded as recommended by the WHO [21]. Distribution of clinico-pathological factors in the study cohort is given in Additional file 1.

### Immunohistochemical staining and histopathological examination

RelA/p65 expression patterns had been determined in 78 of the 81 cases in a previous study [18]. For immunohistochemical detection of HDAC isoforms on tissue samples, prediluted polyclonal rabbit IgG antibody directed against HDAC1 (1:11, Abcam, Cambridge, UK), monoclonal mouse IgG antibody directed against HDAC2 (1:5000, Abcam) and monoclonal mouse IgG antibody directed against HDAC3 (1:500, BD Biosciences) were used on 5 μm paraffin sections. Antibody specificity had already been ascertained in a previous study [22]. Immunohistochemistry was done as previously described [22]. Nuclear staining of HDAC isoforms was scored by applying a semiquantitative immunoreactivity scoring (IRS) system, as previously described [22]. Briefly, intensity of staining as well as percentage of cells stained was evaluated separately. The IRS for each individual case ranging from 0 to 12 was calculated by multiplication of the intensity and frequency scores. Cases exhibiting an IRS from 0-6 were combined in one group (HDAC negative), cases with an IRS of more than 6 were combined in a HDAC positive group. In addition, the patients were grouped according to their overall class I HDAC expression pattern (all three isoforms negative *versus *one or two isoforms positive *versus *all three isoforms positive). Staining of tissue slides was evaluated by an experienced pathologist (WW) who was blinded towards patient characteristics and outcome.

### Statistical evaluation

Statistical analyses were carried out with SPSS 15.0 and GraphPad Prism 4.0. The significance of correlations between HDAC isoform staining patterns and clinico-pathological data was assessed by Fisher's exact test and χ^2 ^test for trends. The correlation of the expression of single HDAC isoforms with each other as well as with cytoplasmic and nuclear RelA/p65 was determined by Spearman rank order correlation (raw scores) and χ^2 ^test for trends test (grouped).

Survivor curves were estimated by the Kaplan-Meier method. Differences in survival were assessed by a log rank test. Statistical analysis of transcription factor assays was done by columnar t-test. P-values < 0.05 were considered statistical significant.

### Cell lines

The human epithelial pancreatic cell line PANC-1 was obtained from ATCC and cultured in Dulbecco's modified Eagle's medium supplemented with 10% heat inactivated fetal bovine serum and 1 mM glutamine.

### RNA interference

Predesigned RelA/p65 siRNA duplexes were purchased from Qiagen (Hilden, Germany; target sequence: AAG CAT TAA CTT CTC TGG AAA, sense: r(GCA UUA ACU UCU CUG GAA A)dTdT; antisense: r(UUU CCA GAG AAG UUA AUG C)dTdT. A nonsilencing siRNA (Control siRNA, Qiagen)) was used as negative control. Cells were transfected with 60 pmol siRNA per well using TransMessenger transfection reagent according to the manufacturer's instructions. Efficacy of transfection was checked after 72 h by immunoblotting.

### Immunoblotting

Total cell lysates from 0,6 × 10^5 ^cells/ml were prepared by lysing cells in 30 μl p38 buffer (62,5 mM Tris HCl, pH 6,8; 2% SDS; 10% glycerol; 50 mM DTT in H_2_O) for 10 min on ice and centrifugation for 15 min at 14 000 rpm. Supernatant was used to ascertain protein concentration using a BCA Protein Assay Kit (PIERCE Biotechnology, Rockford, IL, USA). Protein samples (100 μg) were denaturated at 95°C and subsequently separated on a 12% SDS-PAGE gel. After transfer to nitrocellulose membrane and blocking with I-Block (Tropix, Bedford, MA, USA) for 20 min the samples were probed with Acetyl-H3-antibody (Upstate, Billerica, MA, USA; 1:1000), IκBα antibody (Santa Cruz Biotechnology, Santa Cruz, CA, USA; 1:200), Phospho-IκBα antibody (Epitomics, Burlingame, CA, USA; 1:7000), RelA/p65 antibody (Santa Cruz; 1:250) or antibody against β-actin (Chemicon, Billerica, MA, USA; 1:2500) over night and washed three times with washing buffer (0,1% tween-20 in PBS). Incubation with secondary antibody (Tropix, AP conjugated goat-anti mouse antibody, 1:5000; AP conjugated goat-anti rabbit antibody, 1:5000; 45 min at room temperature) was followed by another wash and subsequent visualisation using CDPstar substrate (Tropix).

### Immunofluorescence

For immunofluorescence, cells were seeded into 4-well-labTEK chamber slides (NUNC, Roskilde, Denmark) at a density of 0,4 × 10^5 ^cells/ml, grown over night and then treated with given concentrations of SAHA (QBiogene, Heidelberg, Germany) or VPA (Sigma-Aldrich, Steinheim, Germany) for 72 h. Stimulation was done by adding 2 μl/ml IL-1β one hour before fixation. Cells were fixed with ice-cold 70% ethanol for 20 min at -20°C and subsequently blocked with serum containing buffer (10% BSA, 1% NGS in PBS) for 30 min. To remove goat serum, chamber slides were washed three times with PBS and probed with primary antibody against RelA/p65 (Santa Cruz Biotechnology, 1:50). Following another wash, cells were incubated with Cy3-conjugated anti-mouse antibody (Jackson ImmunoResearch, West Grove, PA, USA; 1:200) and 2,5 μl DAPI for 30 min at room temperature and finally visualized by confocal fluorescence microscopy (Leica, Solms, Germany).

### NF-κB RelA/p65 transcription factor assay

For measurement of RelA/p65 activity an EZ-Detect Transcription Factor Kit (PIERCE Biotechnology) was used, according to the manufacturer's instructions.

In brief, 20 μg of total protein extract was incubated on a streptavidin-coated 96-well plate with bound NF-κB biotinylated-consensus sequence and detected by specific RelA/p65 antibody and secondary HRP conjugated antibody. After adding a chemiluminescent substrate, binding capability of RelA/p65 was quantified at a luminometer (Labsytems Luminoscan, Helsinki, Finland). To ensure signal specificity, NF-κB Competitor Duplex (PIERCE Biotechnology) as well as specific RelA/p65 siRNA were used as positive/negative controls.

## Results

### Expression patterns of class I HDAC isoforms and nuclear RelA/p65 in pancreatic carcinoma and correlation with clinico-pathological data

To test the impact of class I HDAC expression in human pancreatic carcinoma *in vivo *and to elucidate possible interactions of HDACs and RelA/p65 we evaluated the expression of class I HDACs 1, 2 and 3 by immunohistochemistry and correlated expression data with clinico-pathological features, patient prognosis as well as RelA/p65 expression and nuclear translocation, which has been determined in a previous study in an overlapping cohort [18].

**Figure 1:**
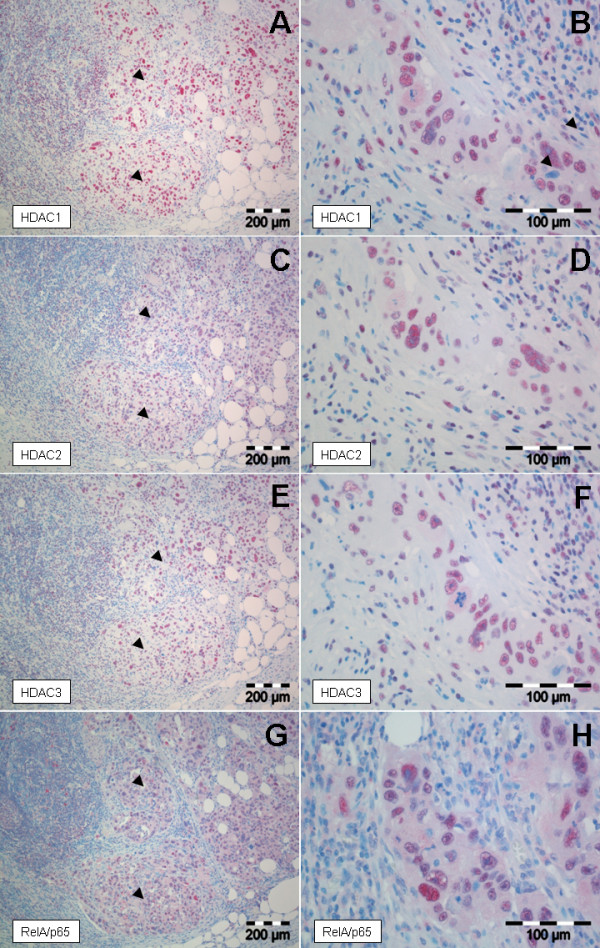
**HDAC1, HDAC2 and HDAC3 as well as RelA/p65 expression in pancreatic carcinomas**. **(A/C/E/G) **Serial sections of a pancreatic ductal adenocarcinoma infiltrating a lymph node. Note strong expression of HDAC1 **(A)**, HDAC2 **(C)**, HDAC3 **(E) **and nuclear positivity for RelA/p65 **(G)**. Magnification ×100. **(B/D/F/H) **Serial sections of a ductal pancreatic adenocarcinoma showing nuclear positivity for HDAC1 **(B)**, HDAC2 **(D) **and HDAC3 **(F)**. Strong expression of the three isoforms is accompanied by strong nuclear translocation of RelA/p65 **(H)**. Weak cytoplasmic RelA/p65 positivity can be seen in this case, as well. Magnification ×400.

Normal acinar cells, normal ductal cells as well as stromal fibroblasts showed occasional weak to moderate positivity for all three HDAC isoforms. Pancreatic adenocarcinoma displayed strong nuclear immunoreactivity for HDAC1 (32%), HDAC2 (63%), HDAC3 (79%) and RelA/p65 (45%) in a considerable number of cases (Figure 1). Some expression was also evident in desmoplastic stroma cells and inflammatory cells. Raw expression scores of HDAC1, HDAC2 and HDAC3 correlated significantly with each other (Table 1), suggesting a shared regulation of these isoforms. High HDAC2 expression was significantly associated with poor tumor differentiation (p = 0.039). No other correlations of HDAC isoforms with clinico-pathological parameters were found (see Additional file 1). Interestingly, we found a positive correlation between RelA/p65 expression and HDAC isoform expression. Categorized grouped HDAC scores significantly correlated with the presence of nuclear RelA/p65 (p = 0.028, data not shown). To further elucidate the strength of the relationship, raw expression scores were compared. Here we found that both cytoplasmic as well as nuclear RelA/p65 positivity was significantly linked with the expression of specific HDAC isoforms, the strength of those correlations were weak to moderate. When the grouped HDAC scores were correlated with RelA/p65 expression, only the relation to nuclear RelA/p65 expression was significant (p = 0.024). The correlation with cytoplasmic RelA/p65 expression showed borderline significance (p = 0.059, Table 1). This supports a functional relationship between HDAC activity and RelA/p65 expression and nuclear translocation (indicating activation). In contrast to nuclear RelA/p65 expression [18] expression of HDAC isoforms 1, 2 and 3 did not have prognostic impact in univariate survival analyses (see Additional file 2 and Additional file 3). For the conventional prognostic parameters tumor grade and nodal status a significant correlation with overall survival could be ascertained in our cohort (grade: p = 0.01; nodal status: p = 0.05) (see Additional file 2).

**Table 1:** Correlation of HDAC1, 2 and 3 expression scores (IRS) with cytoplasmic and nuclear RelA/p65 positivity (IRS).

	Nuclear RelA/p65 IRS	HDAC1 IRS	HDAC2 IRS	HDAC3 IRS	HDAC grouped IRS
**Cytoplasmic**	n = 78	n = 78	n = 78	n = 78	n = 78
**RelA/p65**	**r = 0.640**	**r = 0.191**	**r = 0.246**	**r = 0.105**	**r = 0.215**
**IRS**	*p < 0.001*	*p = 0.094*	*p = 0.030*	*p = 0.358*	*p = 0.059*

**Nuclear**		n = 78	n = 78	n = 329	n = 78
**RelA/p65**		**r = 0.213**	**r = 0.138**	**r = 0.132**	**r = 0.255**
**IRS**		*p = 0.063*	*p = 0.229*	*p = 0.249*	*p = 0.024*

			n = 81	n = 81	n = 81
**HDAC1 IRS**			**r = 0.279**	**r = 0.282**	**r = 0.670**
			*p = 0.012*	*p = 0.011*	*p < 0.001*

				n = 81	n = 81
**HDAC2 IRS**				**r = 0.363**	**r = 0.681**
				*p = 0.001*	*p = 0.006*

					n = 81
**HDAC3 IRS**					**r = 0.572**
					*p < 0.001*

### Inhibition of RelA/p65 activity by treatment with SAHA and VPA

Based on the association of class I HDAC expression and nuclear RelA/p65 translocation in pancreatic adenocarcinoma *in vivo *and the fact that RelA/p65 is a putative target of HDIs we wanted to know if this link could be functionally confirmed for pancreatic cancer *in vitro*.

**Figure 2:**
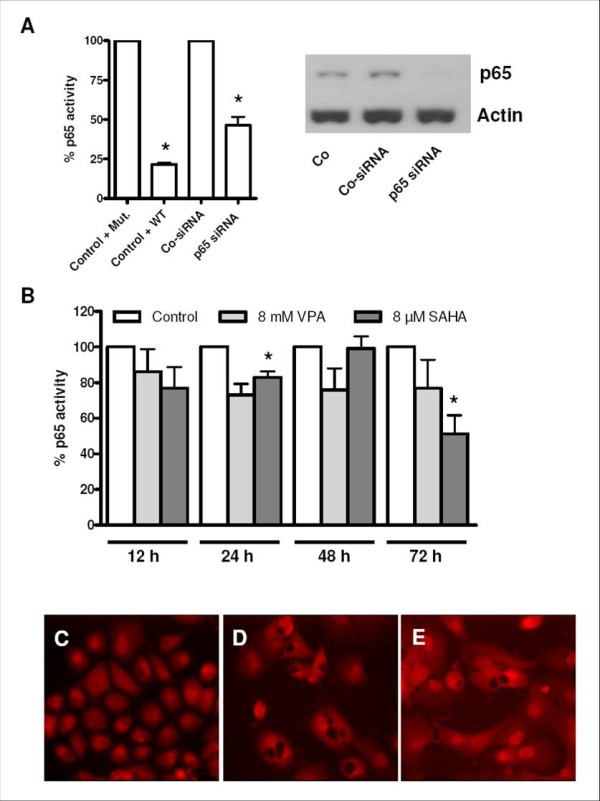
**Effects of HDIs on DNA binding activity and nuclear translocation of RelA/p65 in stimulated PANC-1 cells**. **(A) **Signal specificity of the transcription factor assay was ensured by the use of Wild Type NF-κB Competitor Duplex (WT) (normalized to Mutant NF-κB Competitor Duplex (Mut.)) as well as by specific RelA/p65 siRNA treatment (72 h, normalized to Control siRNA) (*: p = 0.05; columnar t-test); Western Blot showing specific knockdown of RelA/p65 protein expression by RelA/p65 siRNA treatment in comparison with control siRNA and untreated cells. **(B) **RelA/p65 binding activity as measured in a transcription factor assay was markedly diminished in response to 72 h of HDI treatment (*: p = 0.05; columnar t-test, IL-1β stimulated PANC-1 cells). **(C-E) **RelA/p65 specific immunofluorescence. RelA/p65 specific immunofluorescence showed a strong nuclear RelA/p65 staining in response to IL-1β stimulation **(C)**. IL-1β induced nuclear translocation of RelA/p65 was markedly diminished by **(D) **SAHA treatment (8 μM) and **(E) **VPA treatment (8 mM) (72 h).

First, to show that inhibition of nuclear translocation is in fact linked to a decreased activity of RelA/p65, we performed a RelA/p65 specific transcription factor assay which measures the binding activity of the protein. Since the amount of activated RelA/p65 was comparatively low under cytokine absence, RelA/p65 activity was enhanced by stimulation with IL-1β. Stimulation of cells with IL-1β led to an increase of RelA/p65 activity of ~40% in PANC-1 cells. As shown in figure 2A, wild type competitor duplex, but not mutant NF-κB competitor duplex was able to prevent RelA/p65 from binding to the attached consensus sequence. RelA/p65 specific siRNA knockdown, whose efficacy was checked by immunoblotting, reduced RelA/p65 binding activity by ~50% (Figure 2A). As shown in figure 2B, treatment with SAHA and to a lesser degree treatment with VPA resulted in a time-dependent reduction of RelA/p65 activity of up to 50%. In PANC-1 cells, 24 h of VPA treatment decreased RelA/p65 activity by approximately 25%, however, this effect could not be intensified by extension of treatment periods and was not statistically significant. Although lesser effects were also seen for SAHA at shorter time points, a strong and significant RelA/p65 inhibitory effect was only seen after 72 h. As shown in figure 2A and 2B, treatment of cells with 8 μM SAHA for 72 hours was able to diminish RelA/p65 binding activity to the same degree as specific RelA/p65 siRNA knockdown did (~50%). In addition, we found an influence of HDIs SAHA and VPA on the subcellular localization of RelA/p65 by exploratory immunofluorescence analysis. The results of RelA/p65 specific immunofluorescence are shown in figure 2. In untreated (2C) PANC-1 cells we observed a strong signal of RelA/p65 predominantly in the nucleus. Both SAHA (2D) and, to a lesser degree VPA (2E), led to a cytoplasmic retention of the protein after 72 h in stimulated cells.

**Figure 3:**
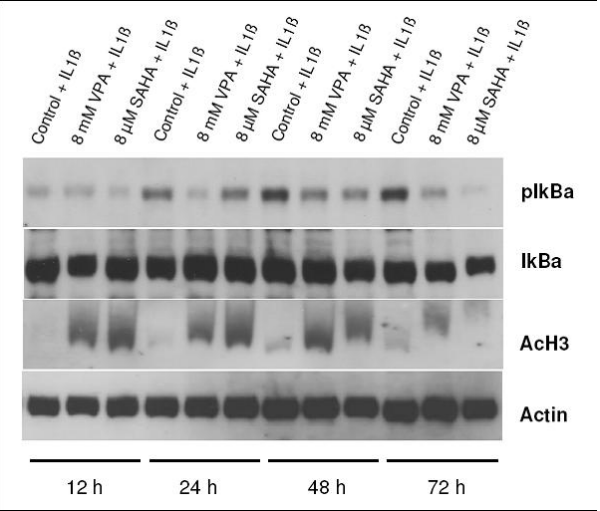
**Effects of SAHA and VPA on IκBα protein level and histone acetylation in PANC-1**. Western Blot showing acetylated histone H3 as well as protein levels of IκBα, p-IκBα and β-Actin in response to SAHA (8 μM) and VPA (8 mM) treatment. HDI treatment led to an increase of histone H3 acetylation and markedly diminished phosphorylation of IκBα.

Both, SAHA and VPA did not affect protein levels of IκBα. Interestingly, phosphorylation of IκBα was notably inhibited by both substances after 72 h of treatment. In contrast, enhanced acetylation of histone H3 could be already observed after 12 h of HDI exposure (Figure 3).

## Discussion

Our study, to our knowledge for the first time, displays a statistically significant *in vivo *correlation between class I HDAC isoform expression in pancreatic carcinoma and the presence of nuclear RelA/p65, a protein known to be a key regulator in pancreatic carcinogenesis. Furthermore, we could demonstrate that HDAC inhibitors are effective in inhibiting nuclear activation and binding capability of RelA/p65 in pancreatic cancer cells.

Although there is strong evidence that aberrant HDAC activity can contribute to the development of cancer, reports on isoform specific expression patterns of HDACs in tumor tissue are sparse [23,24]. In line with previous studies of our group, which focussed on HDAC expression in malignancies of other organs like prostate and colon [25,26], pancreatic tumor tissue displayed a high degree of class I HDAC expression with HDAC3 being the most abundantly expressed isoform. Additionally, our *in vivo *data indicate that a distinct HDAC "high" subgroup is likely to show strong RelA/p65 nuclear translocation/activity, which is of particular interest in pancreatic carcinoma, since activation of the NF-κB pathway is associated with pancreatic cancer development and progression and is an adverse prognosticator for this entity [18]. However, unlike RelA/p65, high class I HDAC expression did not attain statistical significance for overall survival in pancreatic carcinoma in our study, which might be explained by the fact that although we see a correlation between those parameters in our *in vivo *data the correlation is not extremely strong. This suggests that RelA/p65 is only partly regulated/activated by strong HDAC expression. Furthermore, nuclear RelA/p65 as well as cytoplasmic (borderline significant) RelA/p65 positivity was linked to increased HDAC expression when comparing the distributions of raw expression data, which suggests at least in part a transcriptional regulation and not only a post transcriptional modification of RelA/p65 activity by HDACs.

Our survival results are somewhat in contrast to the results reported by Miyake et al. [27], who found a prognostic impact of HDAC1 expression in a moderately large cohort (39 cases) of pancreatic cancers. We cannot exclude that in certain cohorts of pancreatic cancer (e.g. Japanese patients) such a correlation might exist, however, in our Western European cohort which is twice as large (82 cases) as Miyake's cohort, we were not able to confirm these findings and therefore have to conclude that this observed correlation is not universially applicable. Since cut-off values for defining HDAC1 positive/negative cases in Miyake's and our study were different, we repeated our analysis with the cut-offs used by Miyake. However, this did not result in a significant survival difference, either (p > 0.05, data not shown).

The finding that HDAC activity is linked to the activity of RelA/p65 in pancreatic carcinoma *in vivo *could be also confirmed in an *in vitro *cell culture model, where treatment of cells with the HDI SAHA led to a markedly decrease in nuclear translocation and binding activity of RelA/p65. This is in line with previous studies which reported RelA/p65 inhibitory effects of HDIs like Trichostatin A and SAHA in other cell culture systems [28,29]. In contrast, Chen et. al. reported an increase in nuclear translocation and activation of RelA/p65 in response to HDI treatment [30] which suggests that the impact of enhanced RelA/p65 acetylation is dependent on the tumor entity and the HDI used.

The underlying mechanisms and the consequences of RelA/p65 acetylation and deacetylation can't be clarified completely within the scope of this translational work. This should be done in future functional studies. One possible mechanism by which RelA/p65 could be inhibited by HDIs might be the loss of IκBα phosphorylation, which we observe in our *in vitro *experiments and which should lead to an enhanced IκBα accumulation and sequestering of RelA/p65 in the cytoplasm.

Even though treatment with VPA resulted in a partial removal of nuclear translocated RelA/p65 into the cytoplasm in our experiments as well, VPA was less effective in inhibiting DNA binding activity of the transcription factor than SAHA. The different effectiveness of the two HDIs in deactivating RelA/p65 might be explained by the fact that SAHA is known to act on various HDAC isoforms whereas VPA preferentially inhibits class I HDACs [3,31].

Strong antineoplastic effects of SAHA like growth inhibition, induction of apoptosis and cell cycle arrest have been demonstrated in various studies [2,3,7]. Our *in vitro *data on inhibition of RelA/p65 activity indicate that these effects could be partly based on an alteration of the NF-κB signalling pathway. This finding could be of special interest for the development of new treatment strategies for pancreatic carcinoma, since chronic inflammation due to elevated activities of mediators like NF-κB are conductive to pancreatic carcinogenesis [32]. Furthermore, conventional chemotherapeutics like gemcitabine or paclitaxel are often associated with high resistance rates in pancreatic neoplasms, which is partly due to a constitutively activated RelA/NF-κB pathway [15,33]. A combinational treatment with HDIs which are able to inhibit RelA/p65 activity might be of use to overcome such resistances.

## Conclusion

In conclusion, we demonstrated that class I HDACs are overexpressed in pancreatic cancer and that high class I HDAC expression is significantly correlated to nuclear translocation of the transcription factor RelA/p65 in pancreatic adenocarcinoma. The close relationship of class I HDACs and RelA/p65 was confirmed *in vitro*, where nuclear translocation and binding activity of RelA/p65 could be markedly diminished by treatment of pancreatic cancer cells with HDAC inhibitors.

Our data support the assumption that treatment with HDAC inhibitors, either as single agents or in combination with other chemotherapeutics, could serve as a potential approach in the targeted therapy of pancreatic carcinoma.

## List of Abbreviations

DNA: Desoxyribonucleic acid; HDAC: Histone deacetylase; HDI: HDAC inhibitor; IgG: Immunoglobulin G; IL-1β: Interleukin 1β; IRS: Immuno-reactivity score; NF-κB: Nuclear factor 'kappa-light-chain-enhancer' of activated B-cells; PANC-1: Human pancreatic carcinoma, epithelial-like cell line; RelA/p65: NF-κB subunit; SAHA: Suberoyl anilide hydroxamic acid; siRNA: Small interfering RNA; VPA: Valproic acid (2-propylpentanoic acid)

## Competing interests

The authors declare that they have no competing interests.

## Authors' contributions

AL, WW, MD and CD participated in the conceptual design of the study. AB, AN, SD and BM were responsible for pathological data and tissue collection. MB and PN were involved in the clinical and surgical management of the pancreatic cancer patients and for the collection of clinical data. WW and GK did the histological re-evaluation and the determination of staining intensity and extent. WW, CD and JB performed the statistics. AL carried out the cell line experiments and drafted the manuscript. All authors participated in the writing of the manuscript and read and approved the final manuscript.

## Pre-publication history

The pre-publication history for this paper can be accessed here:

http://www.biomedcentral.com/1471-2407/9/395/prepub

## Supplementary Material

Additional file 1 **Expression of class I HDAC isoforms and tumor parameters in the study cohort**. Overall expression of class I HDAC isoforms in pancreatic carcinoma as well as distribution of class I HDAC isoform expression in the study population stratified for selected tumor parameters. In the first row overall distribution of the respective tumor parameters in the study population is listed. Click here for file

Additional file 2 **HDAC expression, clinico-pathological parameters and patient survival**. Influence of HDAC isoform expression and clinico-pathological parameters on patient survival. Click here for file

Additional file 3 **Kaplan-Meier survival curves in dependence of HDAC isoform expression patterns**. Overall survival in dependence of HDAC1 (A), HDAC2 (B), HDAC3 (C) expression as well as in dependence of nodal status (D) and tumor grade (E). P-values were calculated with a log-rank test. Click here for file
