# Supplementary material for: High class I HDAC activity and expression are associated with RelA/p65 activation in pancreatic cancer in vitro and in vivo
Source: BMC Cancer. 2009 Nov 13;9:395. doi: 10.1186/1471-2407-9-395 (PMC2779818; doi:10.1186/1471-2407-9-395)
Supplement: Additional file 1 — Expression of class I HDAC isoforms and tumor parameters in the study cohort. Overall expression of class I HDAC isoforms in pancreatic carcinoma as well as distribution of class I HDAC isoform expression in the study population stratified for selected tumor parameters. In the first row overall distribution of the respective tumor parameters in the study population is listed. [file 1471-2407-9-395-S1.doc]

|  | **All cases** | **HDAC1 *negative*** | **HDAC1 *positive*** | **p-value** | **HDAC2 *negative*** | **HDAC2 *positive*** | **p-value** | **HDAC3 *negative*** | **HDAC3 *positive*** | **p-value** | **gHDAC  *all* *negative*** | **gHDAC *part.* *positive*** | **gHDAC *all positive*** | **p-value** |
| --- | --- | --- | --- | --- | --- | --- | --- | --- | --- | --- | --- | --- | --- | --- |
|  |  |  |  |  |  |  |  |  |  |  |  |  |  |  |
| ***All cases*** |  |  |  |  |  |  |  |  |  |  |  |  |  |  |
|  | 81 (100%) | 55 (67.9%) | 26 (32.1%) |  | 30 (37%) | 51 (63%) |  | 17 (21%) | 64 (79%) |  | 8 (9.9%) | 53 (65.4%) | 20 (24.7%) |  |
| ***Age*** |  |  |  | 0.347^*^ |  |  | 1.000^*^ |  |  | 0.100^*^ |  |  |  | 0.233^+^ |
| ≤65 years | 40 (49.4%) | 25 (62.5%) | 15 (37.5%) |  | 15 (37.5%) | 25 (62.5%) |  | 5 (12.5%) | 35 (87.5%) |  | 2 (5%) | 27 (67.5%) | 11 (27.5%) |  |
| >65 years | 41 (50.6%) | 30 (73.2%) | 11 (26.8%) |  | 15 (36.6%) | 26 (63.4%) |  | 12 (29.3%) | 29 (70.7%) |  | 6 (14.6%) | 26 (63.4%) | 9 (22%) |  |
| ***Tumor stage*** |  |  |  | 0.578^+^ |  |  | 0.420^+^ |  |  | 0.523^+^ |  |  |  | 0.732^+^ |
| T1 | 1 (1.2%) | 1 (100%) | 0 (0%) |  | 1 (100%) | 0 (0%) |  | 1 (100%) | 0 (0%) |  | 1 (100%) | 0 (0%) | 0 (0%) |  |
| T2 | 28 (34.6%) | 17 (60.7%) | 11 (39.3%) |  | 10 (35.7%) | 18 (64.3%) |  | 5 (17.9%) | 23 (82.1%) |  | 3 (10.8%) | 16 (57.1%) | 9 (32.1%) |  |
| T3 | 49 (60.5%) | 35 (71.4%) | 14 (28.6%) |  | 19 (38.8%) | 30 (61.2%) |  | 11 (22.4%) | 38 (77.6%) |  | 4 (8.2%) | 35 (71.4%) | 10 (20.4%) |  |
| T4 | 3 (3.7%) | 2 (66.7%) | 1 (33.3%) |  | 0 (0%) | 3 (100%) |  | 0 (0%) | 3 (100%) |  | 0 (0%) | 2 (66.7%) | 1 (33.3%) |  |
| ***Nodal status*** |  |  |  | 0.801^*^ |  |  | 0.810^*^ |  |  | 0.392^*^ |  |  |  | 0.723^+^ |
| N0 | 26 (32.1%) | 17 (65.4%) | 9 (34.6%) |  | 9 (34.6%) | 17 (65.4%) |  | 7 (26.9%) | 19 (73.1%) |  | 3 (11.5%) | 17 (65.4%) | 6 (23.1%) |  |
| N1 | 55 (67.9%) | 38 (69.1%) | 17 (30.9%) |  | 21 (38.2%) | 34 (61.8%) |  | 10 (18.2%) | 45 (81.8%) |  | 5 (9%) | 36 (65.5%) | 14 (25.5%) |  |
| ***Grade*** |  |  |  | 0.804^+^ |  |  | 0.039^+^ |  |  | 0.134^+^ |  |  |  | 0.198^+^ |
| G1 | 7 (8.6%) | 6 (85.7%) | 1 (14.3%) |  | 5 (71.4%) | 2 (28.6%) |  | 2 (28.6%) | 5 (71.4%) |  | 1 (14.3%) | 5 (71.4%) | 1 (14.3%) |  |
| G2 | 41 (50.6%) | 26 (63.4%) | 15 (36.6%) |  | 16 (39%) | 25 (61%) |  | 11 (26.8%) | 30 (73.2%) |  | 6 (14.6%) | 25 (61%) | 10 (24.4%) |  |
| G3 | 33 (40.8%) | 23 (69.7%) | 10 (30.3%) |  | 9 (27.3%) | 24 (72.7%) |  | 4 (12.1%) | 29 (87.9%) |  | 1 (3%) | 23 (69.7%) | 9 (27.3%) |  |
| ***HDAC1*** |  |  |  | / |  |  | 0.028^*^ |  |  | 0.077^*^ |  |  |  | <0.001^+^ |
| negative | 55 (67.9%) | / | / |  | 25 (45.5%) | 30 (54.5%) |  | 15 (27.3%) | 40 (72.7%) |  | 8 (14.5%) | 47 (85.5%) | 0 (0%) |  |
| positive | 26 (32.1%) | / | / |  | 5 (19.2%) | 21 (80.8%) |  | 2 (7.7%) | 24 (92.3%) |  | 0 (0%) | 6 (23.1%) | 20 (76.9%) |  |
| ***HDAC2*** |  |  |  | 0.028^*^ |  |  | / |  |  | 0.161^*^ |  |  |  | <0.001^+^ |
| negative | 30 (37%) | 25 (83.3%) | 5 (16.7%) |  | / | / |  | 9 (30%) | 21 (70%) |  | 8 (26.7%) | 22 (73.3%) | 0 (0%) |  |
| positive | 51 (63%) | 30 (58.8%) | 21 (41.2%) |  | / | / |  | 8 (15.7%) | 43 (84.3%) |  | 0 (0%) | 31 (60.8%) | 20 (39.2%) |  |
| ***HDAC3*** |  |  |  | 0.077^*^ |  |  | 0.161^*^ |  |  | / |  |  |  | <0.001^+^ |
| negative | 17 (21%) | 15 (88.2%) | 2 (11.8%) |  | 9 (52.9%) | 8 (47.1%) |  | / | / |  | 8 (47.1%) | 9 (52.9%) | 0 (0%) |  |
| positive | 64 (79%) | 40 (62.5%) | 24 (37.5%) |  | 21 (32.8%) | 43 (67.2%) |  | / | / |  | 0 (0%) | 44 (68.8%) | 20 (31.2%) |  |
| ***nuclear p65*** |  |  |  | 0.086^*^ |  |  | 0.026^*^ |  |  | 0.120^*^ |  |  |  | 0.028^+^ |
| negative | 43 (55.1%) | 32 (74.4%) | 11 (25.6%) |  | 20 (46.5%) | 23 (53.5%) |  | 12 (27.9%) | 31 (72.1%) |  | 8 (18.6%) | 26 (60.5%) | 9 (20.9%) |  |
| positive | 35 (44.9%) | 20 (57.1%) | 15 (42.9%) |  | 8 (22.9%) | 27 (77.1%) |  | 5 (14.3%) | 30 (85.7%) |  | 0 (0%) | 24 (68.6%) | 11 (31.4%) |  |

* Fisher’s exact test

+ χ^2^ test for trends

gHDAC: grouped HDAC expression
