# Supplementary material for: High class I HDAC activity and expression are associated with RelA/p65 activation in pancreatic cancer in vitro and in vivo
Source: BMC Cancer. 2009 Nov 13;9:395. doi: 10.1186/1471-2407-9-395 (PMC2779818; doi:10.1186/1471-2407-9-395)
Supplement: Additional file 2 — HDAC expression, clinico-pathological parameters and patient survival. Influence of HDAC isoform expression and clinico-pathological parameters on patient survival. [file 1471-2407-9-395-S2.doc]

|  |  | **Cases** | **Events** | **Mean survival (months)** | **Standard error** | **Log rank (p-value)** |
| --- | --- | --- | --- | --- | --- | --- |
| ***Age*** | ≤65 years | 40 | 32 | 12.4 | 1.8 |  |
|  | >65 years | 41 | 32 | 16.3 | 1.9 | 0.395 |
| ***Tumor stage*** | T1/2 | 29 | 24 | 16.3 | 1.7 |  |
|  | T3/4 | 52 | 40 | 13.1 | 1.9 | 0.970 |
| ***Nodal status*** | N0 | 26 | 16 | 16.3 | 5.9 |  |
|  | N1 | 55 | 48 | 13.8 | 1.5 | 0.049 |
| ***Grade*** | G1/G2 | 48 | 34 | 18.0 | 1.5 |  |
|  | G3 | 33 | 30 | 11.5 | 1.9 | 0.010 |
| ***HDAC1*** | negative | 55 | 43 | 14.7 | 2.2 |  |
|  | positive | 26 | 21 | 13.8 | 2.6 | 0.910 |
| ***HDAC2*** | negative | 30 | 23 | 13.3 | 3.0 |  |
|  | positive | 51 | 41 | 14.7 | 2.8 | 0.585 |
| ***HDAC3*** | negative | 17 | 12 | 20.3 | 2.7 |  |
|  | positive | 64 | 52 | 12.0 | 1.8 | 0.126 |
| ***HDAC groups*** | all negative | 8 | 5 | 20.3 | 2.9 |  |
|  | part. positive | 53 | 43 | 13.1 | 1.5 |  |
|  | all positive | 20 | 16 | 14.7 | 5.7 | 0.395 |
| ***nuclear p65*** | negative | 43 | 27 | 48.5 | 7.6 |  |
|  | positive | 35 | 34 | 15.6 | 1.8 | 0.008 |
