# Supplementary figures and images for: High class I HDAC activity and expression are associated with RelA/p65 activation in pancreatic cancer in vitro and in vivo
Source: BMC Cancer. 2009 Nov 13;9:395. doi: 10.1186/1471-2407-9-395 (PMC2779818; doi:10.1186/1471-2407-9-395)

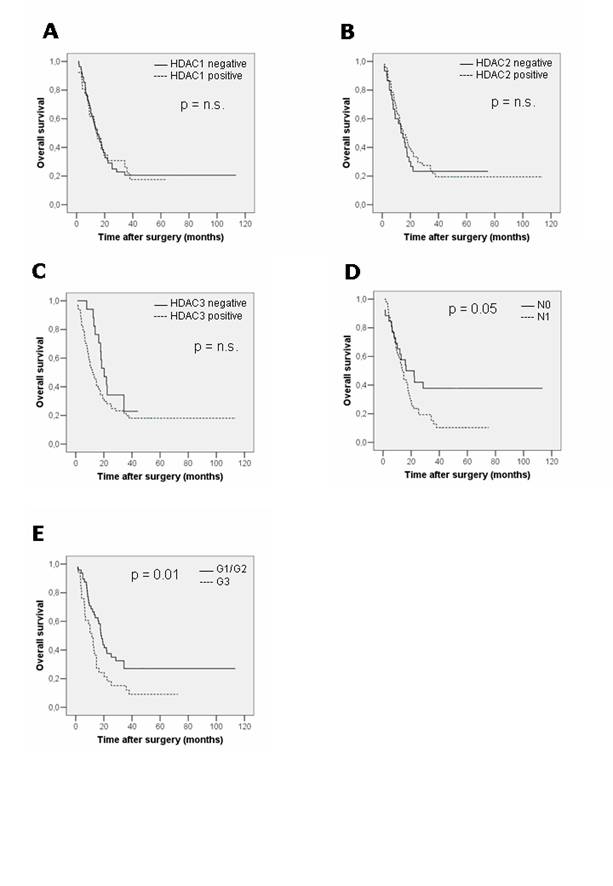

Supplement: Additional file 3 — Kaplan-Meier survival curves in dependence of HDAC isoform expression patterns. Overall survival in dependence of HDAC1 (A), HDAC2 (B), HDAC3 (C) expression as well as in dependence of nodal status (D) and tumor grade (E). P-values were calculated with a log-rank test. [file 1471-2407-9-395-S3.jpeg]
